# Therapeutic Strategies against Biofilm Infections

**DOI:** 10.3390/life13010172

**Published:** 2023-01-06

**Authors:** Sonal Mishra, Amit Gupta, Vijay Upadhye, Suresh C. Singh, Rajeshwar P. Sinha, Donat-P. Häder

**Affiliations:** 1 Laboratory of Photobiology and Molecular Microbiology, Centre of Advanced Study in Botany, Institute of Science, Banaras Hindu University, Varanasi 221005, Uttar Pradesh, India; 2 Department of Microbiology, Parul Institute of Applied Science (PIAS), Center of Research for Development (CR4D), Parul University, Vadodara 391760, Gujarat, India; 3 Pathkits Healthcare Pvt. Ltd., Gurugram 122001, Haryana, India; 4 Department of Botany, Emeritus from Friedrich-Alexander University, 91096 Möhrendorf, Germany

**Keywords:** biofilm, biofilm infections, drug resistance, EPS matrix, nanoparticles, quorum sensing

## Abstract

A biofilm is an aggregation of surface-associated microbial cells that is confined in an extracellular polymeric substance (EPS) matrix. Infections caused by microbes that form biofilms are linked to a variety of animals, including insects and humans. Antibiotics and other antimicrobials can be used to remove or eradicate biofilms in order to treat infections. However, due to biofilm resistance to antibiotics and antimicrobials, clinical observations and experimental research clearly demonstrates that antibiotic and antimicrobial therapies alone are frequently insufficient to completely eradicate biofilm infections. Therefore, it becomes crucial and urgent for clinicians to properly treat biofilm infections with currently available antimicrobials and analyze the results. Numerous biofilm-fighting strategies have been developed as a result of advancements in nanoparticle synthesis with an emphasis on metal oxide np. This review focuses on several therapeutic strategies that are currently being used and also those that could be developed in the future. These strategies aim to address important structural and functional aspects of microbial biofilms as well as biofilms’ mechanisms for drug resistance, including the EPS matrix, quorum sensing (QS), and dormant cell targeting. The NPs have demonstrated significant efficacy against bacterial biofilms in a variety of bacterial species. To overcome resistance, treatments such as nanotechnology, quorum sensing, and photodynamic therapy could be used.

## 1. Introduction

A biofilm comprises a collection of micro-organisms, primarily bacteria, but also fungi, viruses, protozoan, and other microbes. To ensure their survival, bacteria maintain an organized and structured growth and proliferation strategy on any surface. Antimicrobial chemicals, unfavorable temperature and pH conditions, and other environmental variables cause bacteria to assemble into biofilms as a stress response mechanism [1]. The organization of the bacteria in the biofilm, which takes the shape of microcolonies enclosed in the extracellular polymeric substance (EPS) of the matrix, makes it possible for them to survive. By facilitating the movement of nutrients and water, controlling metabolism and tolerance to desiccation, providing resistance to antibiotics and disinfectants, with cell aggregation inducing coordination of virulence factor expression via QS and affecting predator–prey interactions, the EPS matrix holds the bacterial cells together and gives biofilms resilience and versatility [2,3,4,5].

Prior to the discovery of antibiotics, biofilm infections posed a serious risk to human health and were responsible for many illnesses and even fatalities. Numerous antibiotics have been developed since Fleming’s discovery of penicillin in 1928, which are used to treat bacterial infections saving countless lives [6]. Nevertheless, because antibiotics are overused and misused, bacterial resistance and even multidrug resistance have emerged, signalling the start of the “post-antibiotic age” [7]. Many illnesses are exclusively induced by biofilm infections. Due to the novel resistance mechanisms evolved by biofilm infection, it has become a serious and long-term hazard to public health and the economy as a result of the high mortality rate it causes [8]. These infections are treated with a variety of new antibiotics; however, it has become clear that biofilm bacteria frequently exhibit enhanced antibiotic resistance due to the ineffectiveness of antibiotics against biofilm-associated infections [9]. Furthermore, recent research has revealed that the extracellular polymeric matrix (EPM) has little effect on antibiotic dispersion [10]. Another theory is that after adhering to polysaccharides, DNA, and proteins in the biofilm, antibiotics seem to be more biologically inactive or cannot reach the concentrations necessary for effective bacterial eradication. The challenges discussed above highlight the critical need for novel methods of inhibiting biofilm formation and removal. The creation of new antibiotics, however, continues to be far behind the emergence of bacterial resistance. To combat biofilm infections that are multidrug-resistant, new approaches are desperately needed [11]. In this review, we concentrate on a few key strategies for dispersing bacterial biofilm, including targeting the EPS matrix and QS with the hope of causing an active dispersal through outside interference.

The increase in bacterial resistance to antibiotics has prompted researchers to look into various antimicrobial therapeutic strategies that are utilized to create potent antibacterial agents. Transitioning from normal therapy towards high-tech solutions based on nanomaterials might be one of the possibly successful and unquestionable intriguing substance- or biofilm-fighting strategies. The unique properties of nanomaterials have revolutionized a variety of technologies and areas, including medicine. Nanomaterials, which can be tailored to have sizes comparable to biomolecules and bacterial intracellular structures, can be developed as innovative therapeutic approaches. Nanoparticles work by avoiding bacterial defences against drug resistance and preventing the growth of biofilms or other critical processes linked to a bacterium’s potential for antipathy. Nanoparticles have the ability to enter the cell wall and membrane of bacteria and disrupt vital molecular processes. Nanoparticles may exhibit synergy when used with the right antibiotics and aid in preventing the growing global bacterial resistance. Moreover, polymer-based nanoparticles enable the development of a variety of therapeutic goods due to properties like improved biocompatibility and biodegradability [12]. Recent advancements in nanomaterial-based systems present novel strategies to tackle MDR planktonic and biofilm infections, acting as either intrinsic therapeutics or nanocarriers for antimicrobial drugs. Nanocomposites-based therapies, which have the capacity to sidestep established mechanisms associated with acquired drug resistance, are potential weapons against difficult-to-treat biofilm infections. Metal oxide nanoparticles (NPs) such as TiO_2_, CuO, ZnO, and Fe_3_O_4_ and several mixed metal oxides are among the most promising and frequently investigated NPs. In this study, we summarize all the latest evidence on the efficacy of employing metal oxide NPs against biofilms. Furthermore, antibiotics, matrix-degrading enzymes, photodynamic therapy, quorum sensing inhibitors (QSIs), metal nanoparticles, or chitosan derivatives are a few examples of materials that may negatively affect the biofilm formation [13,14]. Finally, we discuss the current state of clinical development of antimicrobial nanoparticles.

## 2. Structural and Functional Aspects of Microbial Biofilms

At interfaces, micro-organisms gather to create polymicrobial aggregates such as mats, films, or biofilms. The matrix can make up more than 90% of a biofilm’s dry mass, whereas the bacteria typically make up less than 10%. The extracellular matrix in which the biofilm cells are embedded is mostly created by the micro-organisms themselves. A self-produced matrix of hydrated EPS serves as the immediate habitat for the micro-organisms in biofilms. The EPS improves biofilm tolerance to antimicrobials and immune cells in addition to providing structural stability and a functioning environment [15,16]. The biofilm matrix retains water and holds the cells together. Because of the wide variety of matrix biopolymers, EPS has been referred to as “the house of biofilm cells” and “the dark matter of biofilms”. The major components of EPS include a blend of polysaccharides, proteins, lipids, and nucleic acids. DNA assembles in the bacterium *Pseudomonas putida* pure cultures and the EPS matrix of activated sludge [17]. Extracellular DNA (e-DNA) in *P. aeruginosa* biofilms is probably derived from complete genomic DNA [18]. Surprisingly, e-DNA was arranged in different patterns and formed grid-like structures in the biofilms of this organism, indicating a structural role for e-DNA. Similarities and differences between the e-DNA and genomic DNA were evident. In the biofilms of an aquatic bacterium, e-DNA formed as a spatial structure producing a filamentous network [19]. The filaments appeared to act as nanowires, allowing the cells to migrate along them. One of the main matrix elements in *P. aeruginosa* biofilms was e-DNA, which served as an intercellular link. Numerous researchers endorsed the idea of stabilizing e-DNA’s function for the biofilm matrix [20]. The QS systems and iron regulation both regulate the release of e-DNA in *P. aeruginosa* [18,20]. The release of genomic DNA, a crucial structural component of *Staphylococcus aureus* biofilms, occurs as a result of *cidA*-controlled cell lysis, which is a critical factor in the development of *S. aureus* biofilms [21]. Biofilms are mechanically stable due to the EPS, which also facilitates their adherence to biotic or abiotic surfaces and creates a cohesive, three-dimensional (3D) polymer network that links biofilm cells and transiently immobilizes them. Intense interactions, such as cell–cell communication and the creation of synergistic microconsortia, are made possible by EPS, which stabilize the biofilm cells and retains them in close proximity; exopolysaccharides, which provide sites for cohesion and adhesion interactions; proteins, which serve as carbon and energy sources; and e-DNA. Furthermore, it is used for the transmission of resistance genes and the main components of EPS, which contributes to the overall establishment of biofilm structure [22]. The genetic make-up of strains, dietary requirements, and stages of biofilm formation all affect how important the EPS matrix is in relation to these factors [23]. The EPS of biofilms is well recognized for serving as a structural scaffold or a shield against hostile conditions [24,25]. The retention of extracellular enzymes results in the generation of a flexible external digestive system that sequesters dissolved and particulate nutrients from the aqueous phase so that they can be used as food and energy sources. It may turn out that the EPS matrix serves as much more than just a biofilm’s adhesive. Instead, it seems to be an extremely complex system that gives the biofilm a mode of life with specific and advantageous characteristics. More details regarding the precise biofilm EPS components, as well as their localization and stability, are likely to be detected. The revolutionary idea of “biofilm as a tissue” referred to EPS as the “glycocalyx” [26]. In this regard, intriguing research is demonstrating that EPS frequently has a distinct macromolecular “honeycomb” structure in a number of microbial communities [27,28]. Biofilms include a set of qualities that are important for life, including three-dimensional structure, adhesion to surfaces, adhesion to other biofilms, adhesion to one another, host defense mechanisms, and a reduction in antimicrobial resistance [29]. Limited diffusion through biofilm matrix causes serial resistance that negatively impacts the permeability of administered antibiotics. It is documented that using mild acid medications in these situations increases the permeability and efficiency of antibiotics [30]. Perhaps as inferred by functional divergence, EPS may be divided into three main categories: Class I includes architectural EPS, which play a role in signal as well as structural regulation. Class II includes protective EPS, which offer defence against physiological and immune response stresses induced by the host, and class III includes aggregative EPS, which play a role in adhesion and biofilm formation.

## 3. Biofilm Architecture

Transforming the expression patterns of cell surface molecules, virulence factors, and consumption pattern routes that enable growth under unfavourable conditions are all part of the complicated and dynamic process by which bacteria develop from free-living to multicellular communities [31]. Microbial cells and EPS are combined to form a stable biofilm structure, which has a predetermined architecture and offers the best conditions for the exchange of genetic material between cells. QS is another method through which cells can interact. This method may potentially influence biofilm processes like detachment. The understanding of biofilm architecture is necessary because it affects growth rate, fluid flow, and transfer of dissolved species, such as nutrients, metabolites, and disinfectants, across the biofilm/fluid interface and surface architecture [32]. Biofilm architecture may seem to be preferred in a state of needful/poor nutrient supply [33]. The total chemical exchange, biofilm permeability, flow resistance in constrained flow routes, and cell attachment/detachment and sloughing may all be assumed to depend on the surface architecture for a given surface density of biofilm biomass, which may affect a variety of practical applications. The development of biofilms and obstruction of downstream flow pathways are further impacted by detachment and sloughing. The method used to handle growth-induced stresses is particularly important for the establishment of surface architecture [32]. A measure of how frequently deviations occur over distances combined with the divergence from the average surface is known as the fractal dimension. While applying fractal dimensionality to biofilm height and length is undoubtedly helpful, comparing that fractality to other dimensions like height and width enables more sophisticated metrics for figuring out how architecture responds to environmental cues, including flow [34].

## 4. Emerging Issues of Biofilm Resistance

### 4.1. Antibacterial and Multidrug Resistance

There are many different environmental niches where biofilms can grow, including freshwater rivers, rocks, deep-sea vents, and hydrothermal hot springs. Bacterial biofilms are the cause of over 80% of chronic and recurrent microbial illnesses in humans [8]. Cells in biofilms benefit from a genetic adaptation by becoming more resistant to antibiotics and adapting to their environment. Antibiotic resistance is facilitated by the production of multidrug resistance genes by alterations in the outer membrane proteins of the cells within biofilms. For instance, enhanced beta-lactamase expression brought on by imipenem in *P. aeruginosa* biofilms is associated with high levels of imipenem resistance. In biofilms, piperacillin can similarly promote the expression of beta-lactamases. However, it does so at a lower level than imipenem. The primary cause of biofilm persistence in chronic infections is a combination of enhanced beta-lactamase expression and other protective features of the biofilm growth mode [35]. The acquisition of multidrug resistance genes through horizontal gene transfer is the other way by which biofilm cells become resistant to antibiotics. This process aids in the evolution of the cells that make up biofilms [36]. This horizontal transmission between the cells in biofilms is significantly impacted by QS [37]. There is evidence to support the idea that biofilms have evolved these defence mechanisms as a general stress response that prompts the bacteria within the biofilm to respond to possible environmental changes [36]. Innovative tactics that target these systems must be created to combat biofilms.

The emergence of multidrug-resistant strains, which result in untreatable outbreak incidences in hospitals, may be caused by resistance to antimicrobial and biocides, including antiseptic and disinfection chemicals. The phenomenon of multidrug resistance has led to many pathogenic strains being resistant to antibiotics, and some have developed resistance to several antibiotics and chemotherapeutic drugs. The accumulation of genes that each code for resistance to a particular agent on resistance plasmids or transposons, as well as the activity of multidrug efflux pumps, each of which can secrete more than one drug type, are the two main causes of multidrug resistance in bacteria. Numerous pathogens are involved in chronic infections, including *Streptococcus pneumoniae* and *Haemophilus influenzae* in chronic otitis media, *P. aeruginosa* in cystic fibrosis pneumonia, and enteropathogenic *Escherichia coli* in recurrent urinary tract infections that are linked to the development of biofilm. For hospitalized patients, infection with strains that are multidrug-resistant is a serious problem. Infections linked to indwelling medical devices along with native biofilm infections of host tissue are two examples of where the biofilms may develop on abiotic surfaces [38,39].

The biofilm that develops on medical implants such as heart valves, catheters, contact lenses, joint prostheses, intrauterine devices, and dental units can lead to infections of the urinary tract and bloodstream. The only way to treat these infections is to remove the implants, which raises the expense of treatment and presents additional stress for patients [40]. Bacterial biofilms are a critical worldwide health concern because they may withstand medications, host defense mechanisms, and other external stimuli, which leads to persistent chronic infections [41,42]. Recent experimental studies have shown that the presence of two or more species increases resistance and virulence when compared to single-species infections, despite the fact that the effect of bacterial population and interspecific interactions on the severity and treatment of chronic infections is unknown.

Biofilms not only shield micro-organisms from changes in pH, osmolarity, lack of nutrition, mechanical stress, and shear forces but also prevent bacterial biofilm communities from being accessed by antibiotics and the immune cells of the host [40,43,44,45]. As a result, the biofilm matrix gives bacteria an added level of resistance, enabling them to not only withstand harsh conditions but also develop a resistance to antibiotics. This in turn, causes the emergence of infections caused by harmful bugs such as multidrug-resistant, extensively, or completely drug-resistant bacteria. In the development and regulation of *Mycobacterium tuberculosis* biofilm, mycolic acids as well as DNA play a crucial role [46,47,48]. The finding of biofilms in *M. tuberculosis* suggested that the infection associated with clinical biomaterials and prosthetic joints in particular, and the removal of these biomaterials was essential to managing these infections; otherwise, it could lead to the development of antibiotic resistance [49,50]. Antibiotic resistance manifests in biofilm communities due to a number of factors, including the antibiotics’ sluggish or ineffective penetration into the biofilm. The biofilm changed the chemical milieu and a biofilm’s subpopulation of microbes [51,52,53,54,55,56]. These processes, along with the well-known conventional resistance mechanisms, are the results of the multicellular nature of biofilms, which favours antibiotic resistance biofilm communities and results in the failure of treatment strategies [57].

Antibacterial resistance is a problem for modern medicine, as antibiotic efficiency has decreased due to the spread and emergence of resistant micro-organisms in biofilm. Antimicrobial resistance kills an estimated 700,000 people per year [58]. Bacterial species and strains that are resistant to nearly all known antibiotics have emerged as a result of mutations caused by antibiotic misuse in agriculture and veterinary care. The presence of bacteriostatic or bactericidal antimicrobial agents permits the growth of resistant micro-organisms at a concentration that typically inhibits growth. The minimum inhibitory concentration (MIC), which is the lowest concentration at which an antimicrobial drug inhibits microbe growth, is used to assess resistance in planktonic cultures. Resistance can be intrinsic, depending on a cell’s innate characteristics and wild-type genes, and frequently results in mutations or exchange of genetic elements that are resistant to antibiotics [59,60]. These results open new fields of study that investigate how biofilms grow and how antibiotic resistance develops. These prospective targets may aid in the formation of alternative treatments for drug-resistant diseases.

### 4.2. Biofilm Maturation, Dispersal, and Detachment

Biofilm maturation includes disruptive mechanisms that create channels in the biofilm structure and adhesive processes that bind bacteria together while they are proliferating. The 3D structures produced during biofilm maturation, in which the EPS matrix functions as a multifunctional and protective substratum, enable the emergence of a variety of chemical and physical microhabitats, where micro-organisms predominate within polymicrobial and social interactions. Microbial cells start to spread out naturally during the stage of transforming the sessile biofilm into a motile form. However, when mechanical force is applied, bacteria that do not produce extracellular polysaccharides disperse immediately into the surrounding environment. Several saccharolytic enzymes are produced by microbial communities during the dispersion process, allowing surface bacteria to enter a new environment for colonization. *E. coli* produces N-acetyl-heparosan lyase, *S. equisimilis* hyaluronidase, and *P. aeruginosa* and *P. fluorescens* alginate lyase. When microbial cells upregulate protein expression, flagella emerge, which then enable bacteria to move to a new area and aid in disease transmission. Disruption of established or growing biofilms that manifest can be done in a variety of ways, including physical removal, the destruction or remodeling of the EPS matrix, focusing on the development of pathogenic microenvironments due to low pH or hypoxia, and social interactions in polymicrobial biofilms, as well as the elimination of dormant cells. Detachment of the biofilm may occur for biological reasons. It has been found that sometimes a sudden nutrient depletion may be followed by sloughing events, in which biofilm fragments may separate at varying rates, depending on the biofilm’s pace of growth. This opens up many therapeutic intervention opportunities [16,61].

### 4.3. The Mechanism of Drug Resistance in Biofilms

The most important survival benefit conferred by the biofilm mode of life for microbes is the occurrence of antibiotic resistance. Unexpectedly, EPS matrix free-living planktonic analogues carry metabolic waste products through water channels, and biofilm bacteria are antibiotic-resistant multiple times more than nutrient-free bacteria. The two primary causes of infectious biofilm resistance to antibacterial drugs are inadequate antibacterial penetration into biofilms and inherent antibacterial resistance. Poor penetration is caused by reduced antimicrobial diffusion and adsorption on the self-produced EPS protective matrix. Serious and chronic illnesses, including cystic fibrosis, osteomyelitis, pneumonia, and meningitis are brought about by biofilms of pathogenic micro-organisms such as *P. aeruginosa* and *S. pneumonia* [62]. A variety of slow-growing, starving persister cells are produced as a result of nutrient stress in biofilms.

In contrast to the surface layers of antibiotic-susceptible cells, persister cells, which form the internal core of biofilms, are highly resistant to antibiotics [62]. As a result of extensive and unregulated antibiotic usage, several antimicrobial-resistant organisms have emerged, including vancomycin-resistant *Enterococcus*, methicillin-resistant *S. aureus* (MRSA), and multidrug-resistant *Acinetobacter*. There is a need for additional investigations into the specific molecular processes underlying antibiotic resistance, and the development of efflux pumps and pathways that give protection against oxidative stress. Components of EPS, the heterologous method of subpopulation generation, and the suppression of diffusion reactions have all been implicated in the chemical volatilization, precipitation, chelation, and modification of antimicrobial drugs [2,63]. Additionally, certain micro-organisms develop antibiotic resistance by metabolizing mucoid exopolysaccharides into the alginate exopolysaccharide [64]. Multiple factors, including substance delivery, persistent cells, high cell density, an enormous number of resistant mutants, molecular exchanges, and efflux pumps, contribute to biofilm resistance to various antimicrobials. Multiple resistance mechanisms, such as constrained medication absorption, increase biofilm resistance.

## 5. Role of Quorum Sensing in Biofilm Development

Genes involved in quorum sensing (QS) signalling are exploited to regulate the formation of biofilms. A range of inhibitors and substances can interfere with the QS signalling cascade and be utilized as an alternate form of therapy for infections caused by biofilms. Free-living bacterial cells can survive as multicellular organisms via QS mechanisms, if they achieve a specified density and create cell-to-cell connections via the production of small signalling molecules. These signals have a variety of consequences on the genetics and physiology of bacteria [65]. Numerous environmental variables regulate QS-mediated biofilm function, including pH, nutrient availability, and signal flow rates [66]. Signalling molecules bind to receptors on recipient microbial cells, causing several genes to be activated, including those that are responsible for the manufacture of these molecules, and that is how QS works [67]. Both Gram-positive as well as Gram-negative bacteria are known to have a wide variety of QS signalling molecules [68]. “N-acyl homoserine lactones (AHLs), oligopeptides (cyclic thiolactone), hydroxyl-palmitic acid methyl ester furanosyl borate (AI-2), and methyl dodecanoic acid are the primary components of these signals” [65]. Gram-negative bacteria employ diffusible AHLs to cross cell membranes and attach to recipient cell regulatory proteins. On the other hand, Gram-positive bacteria rely on peptide-based sensing systems that count for membrane-bound histidine kinase receptors. Other QS signalling chemicals include quinolone and methyl dodecanoic acid [65]. Through eukaryotic-like Ser/Thr kinase, *B. subtilis* and *S. aureus* control biofilm formation. The phosphorylation of the GroEL chaperone, which is crucial for the development of biofilms in pathogenic *Mycobacterium* and *Streptococcus*, is controlled by PrkC in *Bacillus anthracis*. Numerous pathogenic micro-organisms possess genes for Ser/Thr kinases that are similar to those found in eukaryotes, indicating the relevance of these enzyme systems involved in regulating the biosynthesis of signalling molecules and regulating the biofilm mode of life [69]. QS has been found in eukaryotic micro-organisms *Candida* and *Histoplasma*, as well as subsequently in viruses, in addition to bacteria [68]. Gram-positive and Gram-negative bacteria have an AI-2 autoinducer-based QS pathway found in *V. harveyi* and are engrossed in interspecies communication [65].

## 6. Nanocomposites-Based Therapeutics against Biofilm Infection

Nanocomposites-based medicines, which have the ability to circumvent established processes linked to acquired drug resistance, are prospective weapons against difficult-to-treat biofilm infections. Additionally, nanoparticles’ distinct shape and physical characteristics enable them to target biofilms and treat resistant illnesses. In this review, we highlight the common ways that nanomaterials can be employed to combat bacterial infections brought about by biofilms and acquired antibiotic resistance. When compared to their bulk counterparts, materials at the nano-scale have exclusive physicochemical properties, such as size, shape, and surface. Nanomaterials’ distinctive qualities have revolutionized several technologies and sectors, including medicine. Nanomaterials can be created as novel treatment modalities since they can be made to have sizes equivalent to biomolecules and bacterial intracellular structures [70]. Biofilm-associated resistance, which exacerbates the treatment challenge when bacteria are present in biofilms, frequently necessitates physical removal of the biofilm by rigorous debridement, for example, together with large dosages of antibiotics [71]. These tactics may lead to expensive and time-consuming procedures that may have harmful side effects. Recent developments in nanomaterial-based systems provide new ways to combat MDR planktonic and biofilm infections, serving as either intrinsic therapies or as nanocarriers for antimicrobial medicines [72]. The therapeutic action of nanomaterials is influenced by their distinctive physicochemical characteristics, such as size, shape, and surface chemistry. In comparison to traditional antibiotics, nanomaterials may be less likely to induce resistance and can circumvent existing defense mechanisms [73]. Together with the aforementioned information, nanotechnology offers a new set of tools for developing MDR infection treatment options. Here, we explore the characteristics and features of therapeutic efficacy, shedding light on how nanomaterials could be modified to enhance their effectiveness against planktonic and biofilm infections.

## 7. Nanoparticle (NP) Interaction with Biofilms

**Figure 1:**
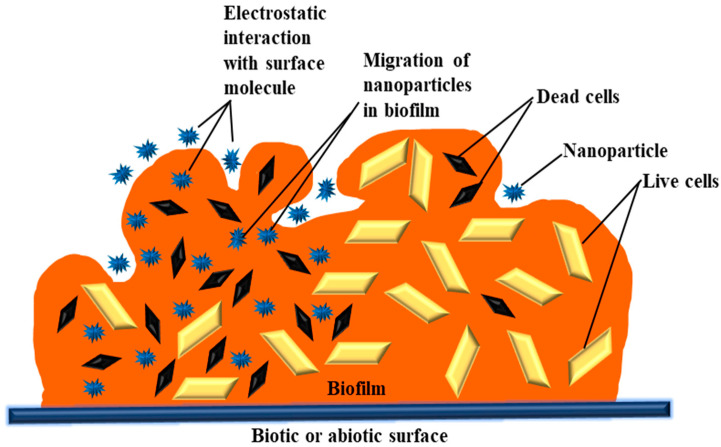
Physicochemical interactions between metal oxide nanoparticles (NPs) and biofilm.

The EPM of biofilms is heterogeneous in terms of the physicochemical properties of a structure composed of numerous polymer molecules carrying an electric charge [74]. Therefore, a biofilm may be seen as a three-dimensional filter capable of removing NPs, organic compounds, and ions. Three stages may be thought of in the interaction between NPs and biofilms: (1) transfer of NPs nearby the biofilm; (2) adhesion to the biofilm surface; and (3) mobilization in biofilms (Figure 1). Each stage’s execution is influenced by a number of factors, including the environment, EPM, and, above all, the physicochemical characteristics of the NPs [75]. There are a number of physicochemical interactions that might influence the first attachment of NPs to the biofilms’ outermost surface. Their electrostatic properties play a major role in determining how NPs and biofilm interact. These characteristics are dependent on the NPs’ zeta potential and the biofilm matrix’s charge [76,77,78]. Due to the presence of uronic acid, metal-bound pyruvate, carboxylic acid, residual phosphate, and occasionally sulphate, most bacteria have a polyionic biofilm matrix [79,80]. Electrostatic forces enable this negatively charged matrix to interact with positively charged metal ions and organic molecules [81,82]. Successfully coupled NPs and EPM on the biofilm surface have different rates of deep penetration into the biofilm. Diffusion is assumed to be the primary cause of NP mobility and penetration inside the biofilm. Under these circumstances, the diffusion of NPs inside the biofilm may be affected by the size of its pores, the existence of water channels, the charge of the NPs, and the EPM [83,84]. The chemical gradient inside the matrix is determined by the hydrophobicity of the surroundings. In the water-containing EPM pore areas, different ion concentrations may exist. Ions and organic molecules move and disperse through these pore spaces throughout the biofilm. This suggests that the spacing between EPM pores may be particularly important in this process. However, this variation on a nanometer scale is not well understood and defined [85,86]. Thus, the charge, size, and composition of the particles, as well as the composition and structure of EPM, will all have a significant role in NP penetration and movement within the biofilm. The exact nature of this connection is yet very much unexplored.

## 8. Metallic NPs’ Impacts on Biofilm by Antifouling

**Figure 2:**
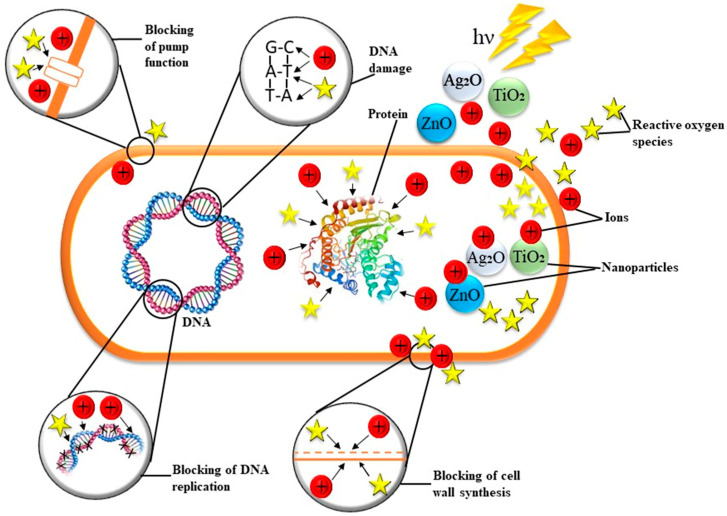
Metal oxide nanoparticle effects on bacterial biofilm. The brown line represents the cell surface (cell wall and cell membrane).

There are three well-known methods that NPs operate to inhibit bacterial growth: (1) mechanical cell wall damage caused by electrostatic interaction, (2) the production of reactive oxygen species (ROS) causing oxidative stress, and (3) metal cation leakage causing disruption of protein functioning and cell structures; all are shown in Figure 2 [72]. They participate in various crucial biological processes, including hydroxylation, electron transport, and redox reactions, and they also form coordination bonds [87]. Metallic NPs contain the metal in a neutral state, making passage through the cell membrane improbable. Nevertheless, it has been shown that metal nanoparticles (NPs) slowly release metal ions that can pierce cell membranes and interfere with intracellular function and processes [88]. Typically, patterns of bactericidal activity resemble those of eukaryote cytotoxicity rather frequently. As a result, the majority of effective bactericidal drugs are poisonous to mammalian cells [89]. The most efficient antibacterial metal oxide at the moment is ZnO, which has efficacies similar to Ag [90]. When exposed to UV radiation, ZnO-NPs become highly bactericidal due to photocatalysis [90]. Here, the NP shape can affect effectiveness. As an illustration, ZnO nanorods were shown to be more effective antimicrobials than nanospheres. The induction of oxidative stress, mostly through the generation of ROS during NP electromagnetic irradiation, is currently regarded as the primary mechanism of bacterial cell death induced by NPs [91]. The production of ROS causes the cell to undergo oxidative stress, which ultimately leads to cell death. Most of the time, cation release is also significantly linked to ROS generation. Fe_2_O_4_ NPs, in particular, release Fe^2+^ ions, which, when combined with hydrogen peroxide, create ROS (Fenton reaction) [92]. Additionally, copper ions can seriously harm nucleic acids and interfere with metabolic processes [93]. It is believed that following copper’s specific binding to DNA, numerous cyclic redox reactions produce OH^−^ radicals close to the binding site, damaging nucleic acids in several ways. However, copper oxidative damage to genetic material in some microbes may occur via Fenton processes [94].

In contrast to zinc oxide NPs, which under UV or visible light create H_2_O_2_ and hydroxyl radicals (OH^−^) but not superoxide radical O_2_^−^, calcium and magnesium oxides may form the superoxide radical O_2_^−^ [95,96]. Because of their charges or reactivity, OH^−^ and O_2_^−^ molecules cannot pass a cell’s membrane [97]. H_2_O_2_ may cross bacterial cells and cause cell death, and persist on the cell surface [98]. Copper oxide nanoparticles (NPs) may create all four varieties of reactive oxygen. Therefore, CuO NPs have strong effects against biofilms and are sufficiently hazardous to bacteria. When exposed to light, TiO_2_ NPs can create electron-hole pairs, resulting in a cascade of oxidation-reduction events on the surface of TiO_2_ and the formation of ROS for subsequent reactions [99,100]. TiO_2_ NPs prevent the oxidation of lipids in membranes, DNA damage, oxidation of nucleotides and amino acids, and bacterial proliferation prevented by photocatalytic degradation of protein-catalytic sites [100]. Despite the aforementioned, there is evidence that not all ROS creation results in cell death. For example, it has been demonstrated by gene expression analysis that ZnO NPs suppress the expression of genes related to oxidative stress despite the production of ROS. The biomimetic activity and other processes may be the cause of the antibacterial effect [101]. Due to the production of ROS and their propensity to interact with thiol groups (RSH), metal nanoparticles and the antibacterial properties that go along with them may be explained, specifically the protein’s cysteine amino acid (Figure 2). Cell death is the ultimate outcome because it directly impairs the operation of particular enzymes and damages disulfide bridges needed to preserve the integrity of protein folding.

Regardless of ROS products, the release of metal cations can have a devastating effect on cells. Cations might interact with amine and carboxyl groups on microbial cells and sulfhydryl groups on enzymes [102,103]. Due to inaccurate biosynthetic enzyme assembly, the procedure may have an impact on cell metabolism [104]. Everything ultimately results in cell death and disruption of cellular functions. By employing the aforementioned processes, the NPs addressed in this review demonstrate antibacterial characteristics to varying degrees. If necessary, NPs can be placed in the following antibacterial and antibiofilm efficacy hierarchy: CuO-ZnO-MgO-TiO_2_-Fe_3_O_4_-Al_2_O_3_. Some NPs oxides’ physical and antibiofilm characteristics are presented in Table 1.

## 9. Nanoparticle Measures for Biofilm Eradication and Prevention

Compared to planktonic bacteria, micro-organisms encased in biofilms are naturally more resilient to antibacterial treatment and host immunological responses [116]. Some hypothesized explanations for this are decreased antibiotic penetration into the biofilm, inactivation of antibacterial drugs by EPS components, and altered metabolic state of bacterial cells inside the biofilm [2]. Remediation of biofilm infections is a significant problem given the EPS physicochemical complexity, unpredictability, and component interactions [16]. Because biofilm EPS components continue to exist even after bacteria inactivation or death, using only antimicrobial methods is hampered. The residual EPS matrix may make it easier for more bacteria to colonize in the future [16], having serious implications, for instance, in a rat wound model. It was found that the in vivo dispersion of antibiotic-resistant biofilm bacterium led to deadly septicaemia [117]. Therefore, prospective biofilm dispersion agents will need to undergo extensive safety testing and should be used in conjunction with antibiotics to avoid recolonization [16].

The avoidance or reduction of early adhesion (passive technique) and antimicrobial treatments are two major ways to combat undesirable biofilms (active strategy). Other studies of antifouling surface developments are available [118]. Innovative medication delivery techniques are now possible due to nanotechnology. There is a chance to employ nanocarriers to penetrate through the biofilm; for instance, they might be designed to keep the active ingredient from becoming enzymatically inactive or from adhering to the biofilm matrix or other components around the biofilm infection site. In comparison to free antibiotics, antibiotic encapsulation in organic NPs can enhance their antibacterial potency [119,120]. Immobilization of antibacterial and antibiofilm agents in nanomaterials is an alternative technique for addressing payload degradation, poor delivery of water-insoluble chemicals, insufficient drug uptake, excessive drug efflux, and resistance development. Lipid and polymer nanoparticles (NPs) are of high relevance because of their biocompatibility, adaptability, potential as platforms for targeted or triggered release, and capacity to include both lipophilic and hydrophilic medicines. To our knowledge, there are no liposomal medications for treating biofilm infections on the market right now, although several of them are being developed.

Drug-delivery nanoparticles with targeting ligands may promote better interaction between the nanocarrier and specific bacterial cells inside the matrix. In contrast to nonspecific targeting, which relies on charge-based interactions and hydrogen bonding of the nanocarrier with the biofilm, specific targeting is based on targeting ligands that precisely bind to a target molecule inside the biofilm. For instance, the bacterial cell surface was the target of Triclosan-conjugated poly(ethylene) (PEG-PAE) micelles (d = 100 nm) glycol-poly (β-amino esters) [121]. Triclosan is released when bacterial lipases break down the ester-linkage with PAE in a low-pH environment. Compared to free antimicrobial controls, it has been revealed that this targeted distribution of Triclosan increases the antibacterial potency against biofilms containing MDR *Staphylococcus aureus*, *streptococcal* bacteria and *E. coli*. The antibacterial effectiveness of the nanocarrier was pH sensitive. Such an approach facilitates the systematic development of drug carriers to target and treat Gram-positive, Gram-negative, or bacteraemia infections.

It has been demonstrated that several different kinds of inorganic NPs have antibacterial properties. Although several research efforts have been undertaken on the inhibitory impact of AgNPs on bacterial biofilms, the antibacterial activity of both gold [122] and AgNPs [123] has been extensively documented. It is unclear how bacteria biofilms and AgNPs interact with one another. AgNP aggregates were found in the EPS matrix, revealing a reason why eradicating biofilms is ineffective. The results showed that the duration of exposure to NPs (d = 5–150 nm) substantially influenced biofilm detachment, with considerably less biomass detaching following treatment over 8–24 h periods compared to initial exposure. However, the reasons for this effect were not entirely apparent. Due to variations in ionic strength, interactions with complexing chemicals from the EPS, and the delayed transport of silver ions and particles inside the biofilm matrix, the result was partially attributed to NP aggregation. Under AgNP therapy, it was thought that EPS offered physical protection for bacteria. Bacterial biofilms become more susceptible to AgNPs when the loosely bound EPS from such biofilms is eliminated. Targeting the EPS allows for the rupture of the matrix and may increase the cell sensitivity to antimicrobial methods, making it an essential yet underutilized method for controlling biofilms. As ‘carriers’ of EPS matrix disruptors, NPs can play a crucial role, and several strategies have been proposed [118,124].

A possible method is to use constructed NPs as carriers for specific QS inhibitors. When silica nanoparticles (d = 15 and 50 nm) polymerized with -cyclodextrin were put into *Vibrio fischeri* cells, there was a significant reduction in cell-to-cell communication. Cyclodextrin is a nonspecific acyl homoserine lactone (HSL) signalling a molecule binding agent [125]. *Nigella sativa* seed extract was used to make zinc nanoparticles (ZnNPs, d = 24 nm), which showed broad-spectrum QS suppression in *P. aeruginosa*. Elastase, pyocyanin, protease, and alginate production were all significantly decreased. ZnNPs have been shown to be effective at dispersing mature biofilms of *Listeria monocytogenes*, *C. violaceum*, *P. aeruginosa*, and *E. coli,* as well as inhibiting their development at subinhibitory doses [126]. Using enzyme-polymerized NPs or enzyme mimicry are two more intriguing methods for disrupting EPS. This synthetic nano enzyme with DNase-like activity demonstrated strong eDNA cleavage ability, increased stability, and simple recovery. These NPs prevented bacterial adherence and biofilm development on surfaces for extended periods of time. The NPs were also effective in dispersing established biofilms by EPS disintegration. Additionally, the capacity to get rid of enclosed bacteria and remove biofilms was enhanced when used in conjunction with conventional antibiotics [127].

## 10. Investigative Approaches to Eradicate Biofilms

**Figure 3:**
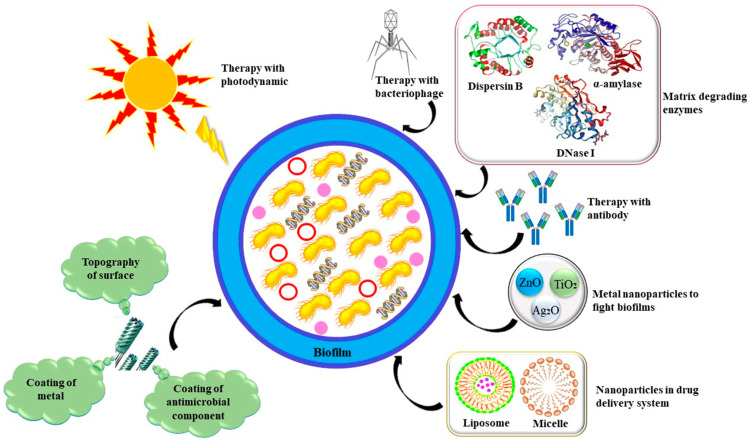
Investigative approaches and anti-biofilm therapeutics to eradicate biofilm infections.

The emanation of antibiotic resistance in biofilm-related infections has resulted in the development of different anti-biofilm agents of prokaryotic and eukaryotic origin. QS modulation has been the focus of anti-biofilm therapeutic research and development since it is critical to biofilm formation and pathogenicity. Antibiotic resistance is becoming more common in many bacterial populations, making it harder to effectively treat biofilm-related infections. Additionally, several conventional antibiotic chemotherapeutic methods are unable to completely remove these bacterial cells, especially those positioned in the centre of the biofilm, which worsens the situation internationally. Therefore, new anti-biofilm compounds and other tactics are necessary to combat antibiotic resistance and biofilm communities. Antibiotics, matrix-degrading enzymes, photodynamic treatment, QSIs, metal nanoparticles, or derivatives of chitosan are a few elements that might affect the structure of biofilm through a variety of processes with different extents of efficiency [13,14]. (Figure 3).

### 10.1. Antibiotics to Be Used with Nano-Based Delivery Approaches

The majority of the modern antibiotics discovered during the last 30 years are synthetic modifications of previously isolated natural forms [128]. Using antibiotic-loaded NPs for bacterial targeting and delivery is an appealing strategy because of its many advantages over traditional formulations, such as improved stability, targeted capabilities, controlled antibiotic release, and higher bioavailability [129]. When compared to free antibiotics or NPs alone, antibiotic-conjugated NPs have stronger antibacterial properties. This shows a synergistic interaction between antibiotics and NPs, showing several antibacterial underlying mechanisms in these molecules [130,131]. In studies, erythromycin, clindamycin, amoxicillin, and vancomycin all showed improved efficacies against *E. coli* and *S. aureus* when combined with AgNPs. This was done without any direct, intentional associations between NPs and antibiotics, although it is possible that the antibiotic and NP accidentally bound together. “bPEI-coated polyacrylic copolymer nanogels have been used to deliver the cationic antibiotics tetracycline and lincomycin to specific locations” [132].

### 10.2. Matrix-Degrading Enzyme

Another efficient anti-biofilm strategy is the use of biofilm matrix-degrading enzymes (such as Dispersin B (DspB), alpha-amylase, and DNase I). When a structural component of the biofilm breaks down, more antibiotics will penetrate the body, increasing their effectiveness. “DspB, DNase I, and α-amylase degrade exopolysaccharides, eDNA, and biofilm matrix, respectively” [14,133]. Many bacteria, including *S. aureus*, *Vibrio cholera*, and *P. aeruginosa*, prevent biofilm formation and destroy established biofilms. As the biofilm matrix is made up of proteins, periplasmic polysaccharides, and DNA, various studies have shown that the breakdown of biofilm components by various enzymes can cause biofilms to lose their structural integrity. The pathogenicity of bacteria that form biofilms is mediated by the exopolysaccharide poly-N-acetylglucosamine (PNAG), which is targeted by dispersin B [134,135]. It inhibits bacterial growth, and in vitro experiments demonstrate that biofilms are practically fully destroyed, indicating that it is an effective agent for eradicating biofilms alone as well as in conjunction with antibiotics. Additionally, it could help to avoid infections. Although various antimicrobial treatments have proved to increase the eradication of mature biofilms, DNase 1 also destabilizes a biofilm by destroying extracellular bacterial DNA (eDNA) [134,136].

### 10.3. Biofilm Dissolution by Photodynamic Therapy (PDT)

**Figure 4:**
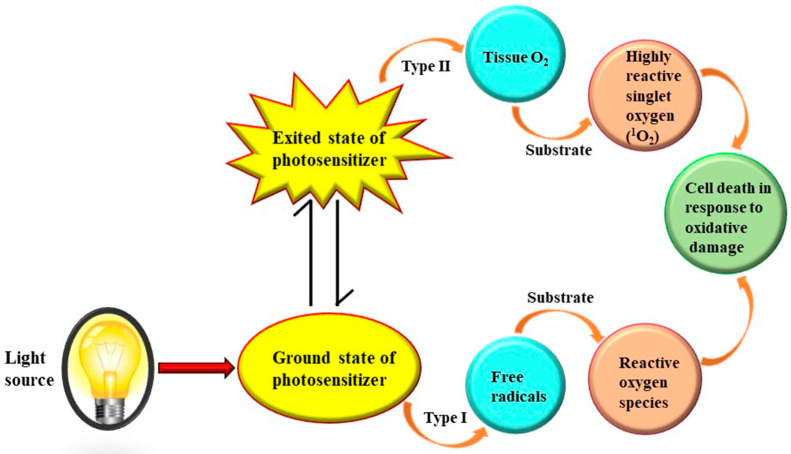
Photodynamic therapy (PDT) consisting of light, photosensitizer, and oxygen for biofilm eradication (Modified from 138).

Photodynamic therapy, a potential antibacterial treatment method, has recently attracted a lot of interest. Photodynamic treatment is based mainly of light, a photosensitizer, and oxygen. Photosensitizers (PSs) can be utilized to generate ROS when exposed to visible or infrared light in the presence of oxygen [137,138]. “Photosensitizers including pyridyl-porphine, phenothiazine dyes, toluidine blue and malachite green are chemical compounds which are absorbed by the targets, bacteria” [134]. These dyes work when exposed to light with a certain wavelength and oxygen. It causes the formation of free radicals as well as highly reactive oxygen species, which damage cells’ DNA and cause eventual death by infecting their plasma membranes [139]. However, excitation occurs when the wavelength range of light and the photosensitizer’s absorbance spectrum overlap [134], (Figure 4). The energy is then transferred to oxygen molecules or other biomolecules depending on the kind of reaction, and photosensitizers transform or convert into an excited triplet state with a prolonged life expectancy.

The Type I process involves the transfer of electrons from the triplet excited singlet state to the target, such as the membrane made of unsaturated phospholipids, which results in the formation of a hydroxyl radical (from water) or the formation of a lipid (from radicals) (illustration shown in Figure 4) [138]. The generated radicals then further interact or mix with oxygen or biomolecules to form hydrogen peroxide, which either causes lipid peroxidation or damages cells by generating ROS [139]. The energy from its triplet state is transferred to a ground state (reduced energy) molecular oxygen in Type II reactions, which produces a quite reactive species called excited singlet oxygen (^1^O_2_). It has the capacity to oxidize cellular macromolecules, including lipids and proteins, which can result in cell death. Both processes can take place simultaneously in a cell, although the Type II mechanism is regarded as a significant APDT pathway. Cells can be disturbed in two ways: through DNA damage or through disruption of cellular organelles. Since DNA is essential for repair and contains information for the generation or development of new organelles and materials, it is a crucial factor in cell death [140,141]. As a result, a significant portion of the micro-biocidal APDT action may be due to an impact on proteins involved in membrane functions, leading to the leakage of the cellular structure outside the cell. Further information and research investigations are needed to determine the effectiveness and efficiency of PDT alone and in conjunction with other antimicrobial treatments.

### 10.4. Quorum Sensing Inhibitors (QSIs)

QS, a kind of bacterial cell-to-cell communication, is directly involved in the growth of many bacterial species’ biofilms. In the biofilm phase, this mechanism can regulate the expression of numerous harmful and virulence genes [142]. QSIs are compounds produced by eukaryotes and/or prokaryotes that can prohibit the QS systems, which can result in a reduction in the activity of efflux pump genes and the breakdown of bacterial biofilms [143]. A number of techniques have been used to destabilize QS, including preventing the synthesis of acyl-homoserine lactones (AHLs), decreasing the activity of the AHL synthase, disrupting and inactivating AHLs, and using several antagonists signalling compounds from competitors [65,144]. As QS regulates many stages of the growth of the biofilm, such as initial colonization, attachment, bacterial aggregation, biofilm maturity, and cell dispersion, suppressing QS will stop the biofilm from developing. As a result, QSIs are used as a therapeutic drug to prevent biofilm infection and inhibit the spread of biofilm [145]. Natural chemicals, synthetic substances, and antibiotics all have the potential to affect the QSI function. Aspirin, meloxicam, and piroxicam are examples of nonsteroidal anti-inflammatory pharmaceuticals that might be used as potential inhibitors to reduce *P. aeruginosa* QS signalling molecules and biofilm growth [146]. Antibiotics with high levels of QSI activity include azithromycin, erythromycin, ciprofloxacin, ceftazidime, gentamicin, ticarcillin, spectinomycin, and streptomycin [147]. In comparison to each molecule alone, the combination of aminoglycoside antibiotics and resveratrol significantly reduces the generation of biological fluids [148]. The synergistic efficacy of curcumin with ciprofloxacin, gentamicin, ceftazidime, and azithromycin on *P. aeruginosa* has been reported [147]. QS signalling molecules demonstrated that the sub-MIC of each of the drugs, both individually and in combination, may significantly slow the growth of the biofilm. In conjunction with gentamicin, zinc oxide (ZnO) nanoparticles, chitosan, and chitosan-ZnO nanocomposite significantly reduced the biofilm formation of *S. aureus* and *P. aeruginosa*, when the bacteria were given MIC and 1/4 MIC doses of the compounds [149]. Therefore, one possible use in the therapy of antibiofilm infections might be the targeting of QS by different anti-QS compounds.

### 10.5. Surface Coating or Modification

The multifunctional surface topography and coating on implants are thought to be an inventive strategy to combat the formation of biofilms. With the current development in the area of surface engineering, several new possibilities are seen. For the development of textured biomaterial nanosurfaces, modern methods, including nanoimprint lithography, colloidal lithography, and the electron beam, are being used [150,151]. The desired result can be achieved without changing the properties of the original material by merely coating the implants. The main techniques employed in the development of antibacterial coatings include preventing bacterial adhesion, obstructing biofilm formation, and inactivating the biofilm. The formation of homogenous thin coatings on various implants is frequently accomplished via electrophoretic, chemical vapour, and physical vapour deposition processes. In recent times, AMPs have been utilized to coat silicon, titanium, glass surfaces, stainless steel, and polystyrene, exhibiting suppression of biofilms [152,153]. Antibiotic coating using several antimicrobial classes, including beta-lactam antibiotics, quinolones, aminoglycosides, and rifamycins is also investigated. Enzymes that impede QS, such as acylase, oxidase, and lactonase, are employed for coating [154]. Rifampin, doxycycline, and clarithromycin were effectively released from methacrylic copolymer films over 21 days while preventing the growth of MRSA biofilms. Clarithromycin and rifampin together were able to kill more than 99.9 percent of MRSA strains. Combined antibiotic treatment offers a strong chance to minimize the antimicrobial resistance seen with individual antibiotics [155]. The coating material’s rapid erosion over time is a disadvantage of the coating method.

### 10.6. Nanocomposites of Natural Polymers (Antibiofilm Metal-Decorated Natural Polymers)

In nanocomposites, chitosan is a typical natural polymer. Its source is chitin, one of nature’s most prevalent polysaccharides, and it shares structural similarities with glycosaminoglycans, which are present in the extracellular matrix of animals. Chitosan has important antibacterial, biocompatible, and biodegradable qualities necessary for antimicrobial action [156], although the precise antibacterial processes behind chitosan’s effect are still unknown. Various investigations have suggested two possible hypotheses: (a) chitosan penetrates the cell wall through pervasion, blocking, or disturbing the bacterium’s physiological function, and (b) a polymer layer formed on the surface, preventing nutrients from entering the cell [157]. In nanocomposites, chitosan and silver function together to increase tensile strength and antibacterial qualities. “In comparison to chitosan without nanoparticles, higher antibacterial effects were shown when combining chitosan, TiO_2_, and Ag at a specific concentration (0.005; 0.003 wt. percent)” [158]. When compared to the control, the combination of chitosan, Ag, and TiO_2_ enhanced oxidative stress and membrane permeability (LDH), which highlighted the necessity for a thorough investigation into the many potential uses for chitosan/Ag/TiO_2_ nanocomposites in antimicrobial applications (Table 1) [158].

## 11. Human Infections Brought about by Biofilm: Pharmacological Intervention

Even yet, every treatment method is employed to combat infections brought about by biofilms. One of them is photodynamic treatment, which has a variety of uses for preventing wound biofilm infection. It is critical to use the therapy correctly to kill and stain bacterial cells while avoiding damage to the patient’s surrounding tissues partly due to the photochemical and photosensitizer reaction [159]. These cutting-edge anti-biofilm techniques tackle the problem of eliminating infections brought about by biofilms. Additionally, QS is important for regulation. Anti-biofilm compounds hamper signalling pathways in both Gram-positive and Gram-negative bacteria. Antibiotic, peptide, enzyme, and polyphenol compounds are all examples of anti-biofilm molecules [159].

## 12. Exploring Nanotechnology-Based Infection Control: Clinical Translation

Right now, it seems improbable but hopefully in the future, these nanotechnology-based antimicrobials chemists have developed will be translated downward clinically and utilized at the bedside for the benefit of patients. These antimicrobials are thought to be promising in combating the threat of incurable infectious biofilms. Most investigations have been in vitro, with fewer moving on to animal models and even fewer to human trials [160]. The development of adequate in vitro and in vivo models demonstrating the efficacy and safety of nanoparticles will lead to clinical acceptance of their application. The transition to human clinical trials is crucial since both in vitro research and animal in vivo investigations have significant limitations. The cost of risk and safety demonstrations is high. Standardized procedures for assessing biocompatibility and nanotoxicology will be necessary for successful clinical translation. Most formulations shown in Table 2 are under clinical development—mainly antimicrobial AgNPs or nanocarriers for antibiotic delivery.

To our knowledge, “the only tobramycin formulation in a Phase-II trial for the treatment of a respiratory tract infection associated with cystic fibrosis is tobramycin encapsulated in 1,2-dipalmitoyl-sn-glycero-3-phosphocholine/1,2-dimyristoyl-snglycero-3-phosphorylglycerol sodium salt liposomes, known as Fluidosomest” [118,161]. Phase III clinical studies for two liposomal nano formulations for regulated antibiotic administration are presently underway. Amikacin’s liposomal version, Arikace, was created to decrease its renal and neurological toxicity and boost its therapeutic effectiveness. Pulmaquin is a formulation based on nanoliposomes developed for the quick and slow release of ciprofloxacin. Although there are still many obstacles blocking the entrance of nanodrugs into clinical settings, such as safety concerns, it is most likely just a matter of time before these cutting-edge therapies address unfulfilled clinical needs [160].

**Table 2:** Clinical studies of nanotechnology-based therapies.

Trade Name	National Clinical Trial Number	Clinical Trial Phase	Nanoparticle Type	Active Component	Infection Type	Antibiofilm Properties against Species:	Reference Clinical Trial Link
**AgTive**	NCT00337714	IV	AgNP	Silver	Central venous catheter (CVC)	*Bacteraemia*	https://clinicaltrials.gov/ct2/show/NCT00337714 [162]
**Silvasorb**	NCT00659204	III	AgNP	Silver	Hemiparesis	Topical infection	https://clinicaltrials.gov/ct2/show/results/NCT00659204 [163]
**Arikace**	NCT01315691	III	Liposomal	Amikacin	Cystic fibrosis	*Pseudomonas Aeruginosa*	https://clinicaltrials.gov/ct2/show/NCT01315691 [164]
**NA**	NCT02726646	II	Polymeric nanoparticle	Doxycycline	Chronic periodontitis	*Porphyromonas gingivalis*	https://clinicaltrials.gov/ct2/show/NCT02726646 [165]
**NA**	NCT01167985	II	Polymeric nanoparticle	Ammonium polyethyleneimine	Root canal infection	*Enterococcus faecalis*	https://clinicaltrials.gov/ct2/show/NCT01167985 [166]
**Pulmaquin**	NCT02104245	III	Liposomal	Ciprofloxacin	Non-Cystic fibrosis bronchiectasis	*Pseudomonas Aeruginosa*	https://clinicaltrials.gov/ct2/show/NCT02104245 [167]

## 13. Conclusions and Future Perspectives

The formation of a biofilm requires complex and dynamic interactions between the surface, micro-organism, and EPS. At the same time, biofilms contain a widespread type of bacteria in nature. Their recalcitrance poses a significant obstacle that conventional antimicrobials have not sufficiently addressed. It has become more difficult to implement eradication strategies due to biofilm spatial heterogeneity in terms of their chemical and microbial composition [168,169]. This study has reviewed a number of extremely novel antimicrobials based on nanotechnology and delivery methods for infection prevention, particularly in the context of improved penetration and targeted antimicrobial administration inside the biofilm. Metal oxide NPs and their nanocomposites are the few approaches that will soon be widely adopted. The cell-free extract of the cyanobacterium, which has strong reducing potential and antimicrobial properties, has been used to effectively synthesize nanoparticles biologically [170,171]. CuO and ZnO nanoparticles [172], which originally exhibited strong antibacterial qualities and were more specifically used to enhance their efficiency among the NPs included in this review, had the strongest antibiofilm properties.

These NPs have achieved considerable success against bacterial biofilms through various bacterial species. Nanotechnology, QS, and photodynamic therapy are a few therapies that may be utilized to overcome resistance. It is significant to emphasize that comprehensive in vivo investigations are required to assess the efficacy of these novel technologies in the biomedical scenario. In therapeutic applications, where it is important to discriminate between harmful and innocuous bacteria as well as host tissue, specificity is a crucial component. The main obstacles to therapeutic usage of nanoparticles still include systemic safety and long-term impacts on the body. Researchers are now establishing their pharmacokinetic profiles to better understand how nanoparticles behave inside the body. A thorough evaluation of NP toxicity and its effects on commensals is also required. Future research should concentrate on eliminating entire biofilms, while simultaneously addressing the EPS matrix and the cells, enhancing therapeutic potential and decreasing toxicity and resistance development.

## Figures and Tables

**Table 1:** Physical features of metal oxide nanoparticles (NPs) and nanocomposite combating biofilm infection.

Nanoparticles (NP)	Physical Size	Description	Antibiofilm Properties against Species:	References
**CuO**	40 nm	Binds to carboxyl and amines groups.	*B. subtilis* *P. aeruginosa* *S. aureus*	[79] [105]
**ZnO**	<100 nm 22 nm	Interferes with ROS production and damages membranes.	*B. subtilis* *E. coli* *P. aeruginosa* *B. subtilis*	[79] [105] [106]
**MgO**	~23 nm	Usually result in the membrane’s destruction.	*E. coli* *S. aureus* *R. solanacearum*	[107] [108]
**TiO_2_**	<100 nm 40~60 nm ~20 nm	N/A Needs photoactivation.	MRSA biofilm *B. subtilis* *P. aeruginosa* *E. coli*	[109] [79] [110]
**Fe_3_O_4_**	10 nm	DNA, proteins, and cell membranes are harmed by oxidative stress.	*S. aureus* *P. aeruginosa* *E. coli* *B. subtilis* (ATCC 6633)	[111] [112]
**Al_2_O_3_**	60 nm	Flocculation, ROS, and particle penetration that is dose-dependent.	*E. coli* *B. subtilis* *P. fluorescens*	[90]
**Ag**	<100 nm ~10 nm	Binds to carboxyl and amine groups. Significant antibacterial capabilities, although it could work less against Gram-positive bacteria.	*B. subtilis* *E. coli* *P. putida* KT2442 *P. aeruginosa*	[79] [90]
**Ag-TiO_2_**	20~34	Increased oxidative stress and membrane permeability.	*B. subtilis* *S. aureus* *E. coli*	[113]
**Au**	8~34	Optical properties.	*S. aureus* *E. Coli*	[114]
**Titanium dioxide**	~50 nm	First phase (inhibition EPS production).	*B. subtilis* *E. coli* MRSA	[79] [110]
**Chitosan oligosaccharide-capped gold**	<65 nm	Sessile cells’ ability to adhere to surfaces was hampered (initial stage).	*P. aeruginosa*	[115]

## Data Availability

Not applicable.
